# Lon/Pim1-mediated degradation of presequence translocase-associated motor components Pam16 and Pam18 in *Saccharomyces cerevisiae*

**DOI:** 10.1042/BCJ20243016

**Published:** 2025-11-04

**Authors:** Yerranna Boggula, Arpan Chatterjee, Gaurav Simaiya, Amita Pal, Akash Srinivasan, Naresh Babu V. Sepuri

**Affiliations:** Department of Biochemistry, School of Life Sciences, University of Hyderabad, Hyderabad, 500046, India

**Keywords:** Lon/Pim1 protease, mitochondria, mitochondrial protein import, presequence translocase-associated motor, *Saccharomyces cerevisiae*, AAA+ (ATPases Associated with diverse cellular Activities)

## Abstract

Mitochondrial protein homeostasis depends mainly on the efficient import and folding of nuclear-encoded proteins, and defects in this process can lead to proteotoxicity, which is harmful to the cell. Mitochondrial chaperones and proteases are essential defense mechanisms that ensure dysfunctional proteins’ proper concentration, folding, and degradation. Lon protease 1 (Pim1 in yeast) is the mitochondrial matrix protease known to prevent protein aggregation by degrading unfolded proteins. Here, we show that two essential components of ATP-dependent presequence translocase and associated motor (PAM complex), Pam18 and Pam16, are specifically targeted for degradation by the proteolytically active Lon/Pim1, both *in vitro* and *in vivo*. Furthermore, overexpression of Pam18 and Pam16 exacerbates the growth defect of the delta *pim1* strain. Hence, our study reveals, for the first time, that components involved in protein import are substrates of Pim1, which could have potential implications for regulating mitochondrial protein import and proteostasis.

## Introduction

Mitochondria contain ~1000 proteins, most encoded by nuclear DNA, translated in cytosol, and imported into the organelle. The outer mitochondrial membrane (TOM complex) translocase is the entry gate for most mitochondrial precursor proteins. Precursor proteins containing cleavable N-terminal presequence targeted to the matrix or inner membrane via the presequence translocase of the inner membrane (TIM23 complex) [1–7]. The presequence translocase-associated motor (PAM) complex, which resides on the matrix side of the inner membrane, is involved in the subsequent translocation and release of precursor proteins into the matrix [8–12]. This motor comprises five essential components: mtHsp70, Tim44, Mge1, Pam16, and Pam18. Mitochondrial heat shock protein 70 (mtHsp70) is the central component of the PAM complex [11,13–15]. mtHsp70 requires other essential components of the PAM complex for its function—Tim44 is a peripheral inner membrane protein that transiently anchors mtHsp70 directly at Tim23 [16–18]. Mge1, a co-chaperone that acts as a nucleotide exchange factor, recycles Hsp70 by exchanging ATP for ADP [19–22][23]. Pam16 and Pam18 are two membrane-bound essential co-chaperones that interact w

ith each other and influence the ATPase activity of mtHsp70 [24–30]. Apart from these five components, Pam17 is identified as a non-essential component of the PAM complex. It is known to specifically organize the Pam16–Pam18 complex and is involved in the initial steps of the translocation process [31,32].

Pam18, a J-protein, is a 168-amino acid protein that contains a J-domain at the C-terminal, which localizes to the matrix, and an N-terminal domain in the intermembrane space (IMS). This IMS domain stabilizes Pam18’s association with the translocon by interacting with Tim17 and Tim23. Additionally, the J-domain of Pam18 interacts with Pam16, a J-like protein. It is a 149-amino acid protein that interacts with the translocon indirectly through interaction with Tim44. Hence, a multifaceted association of Pam18 with the translocon occurs through direct interactions in the IMS and indirect interactions in the matrix. Pam16, unlike Pam18, is not a typical J-domain protein. It has a J-like domain that has structural and sequence similarity to the J-domain of Pam18 but is incapable of stimulating the ATPase activity of Ssc1 [33]. Pam16 and Pam18 form a stable heterodimer by interacting via their J and J-like domains. This interaction is critical for protein translocation [30].

Mitochondria contain an elaborate network of protein quality control [34,35] that includes molecular chaperones and proteases. Mitochondrial chaperones and proteases residing in the mitochondria seem to act as a first layer of quality control within the organelle [36–39]. Failures in the internal mitochondrial proteolysis lead to an imbalance in the cellular homeostasis. The ATPases Associated with diverse cellular Activities (AAA+) superfamily comprises a broad group of ATP-dependent molecular machines involved in diverse cellular functions such as protein remodeling, DNA replication, and membrane dynamics. AAA+proteases are a specialized subset that couple ATP-dependent substrate unfolding with proteolytic degradation, playing a key role in mitochondrial protein quality control. Although several proteases are present in mitochondria, four intrinsic AAA+proteases are the key players that perform most quality control tasks: i-AAA, m-AAA, Lon, and ClpXP protease [40–42]. These proteases are conserved from yeast to humans except for ClpXP, which is not present in yeast. Lon protease is crucial in maintaining mitochondrial homeostasis by degrading the unwanted and damaged proteins in the mitochondrial matrix [43–45]. Apart from its primary function as a protease, it is also involved in mitochondrial DNA (mtDNA) maintenance and is known to have chaperone-like functions [46–49]. The highly evolutionarily conserved Lon with homologs present in all kingdoms [50]. The Lon protease homolog called Pim1 in yeast has been shown to degrade misfolded or unassembled proteins. Pim1 degrades not only reporter proteins but also endogenous proteins such as Mitochondrial Ribosomes Proteins(MRPs) and Oxidative Phosphorylation (OXPHOS) subunits, and is also required to maintain mitochondrial genome integrity [51,52]. A quantitative analysis of the mitochondrial proteome has demonstrated that proteins with a labile tertiary structure were primary targets of Pim1-mediated degradation, indicating that Pim1 acts as the primary quality control protease in the mitochondrial matrix [53,54]. Moreover, during oxidative stress conditions, the mitochondrial enzymes dihydroxy acid dehydratase and aconitase were strongly susceptible to degradation by Pim1/Lon, presumably because they become unstable after covalent oxidative modification of their Fe/S cluster cofactor [55–57]. Studies show that Lon has chaperone-like activity apart from its well-established protease activity. TIM23 subunit and mtHsp70 appear to closely co-operate with Lon protease in its chaperone-like functions [38,58].

To understand Pim1/Lon-mediated protein homeostasis in mitochondria, we examined the role of Lon protease in the turnover of mitochondrial import components. In this study, we show that two essential PAM complex proteins, Pam16 and Pam18, are substrates for Lon/Pim1 protease, not by other mitochondrial matrix localized proteases.

## Results

### Lon protease interacts and degrades Pam16 and Pam18 *in vitro*

**Figure 1:**
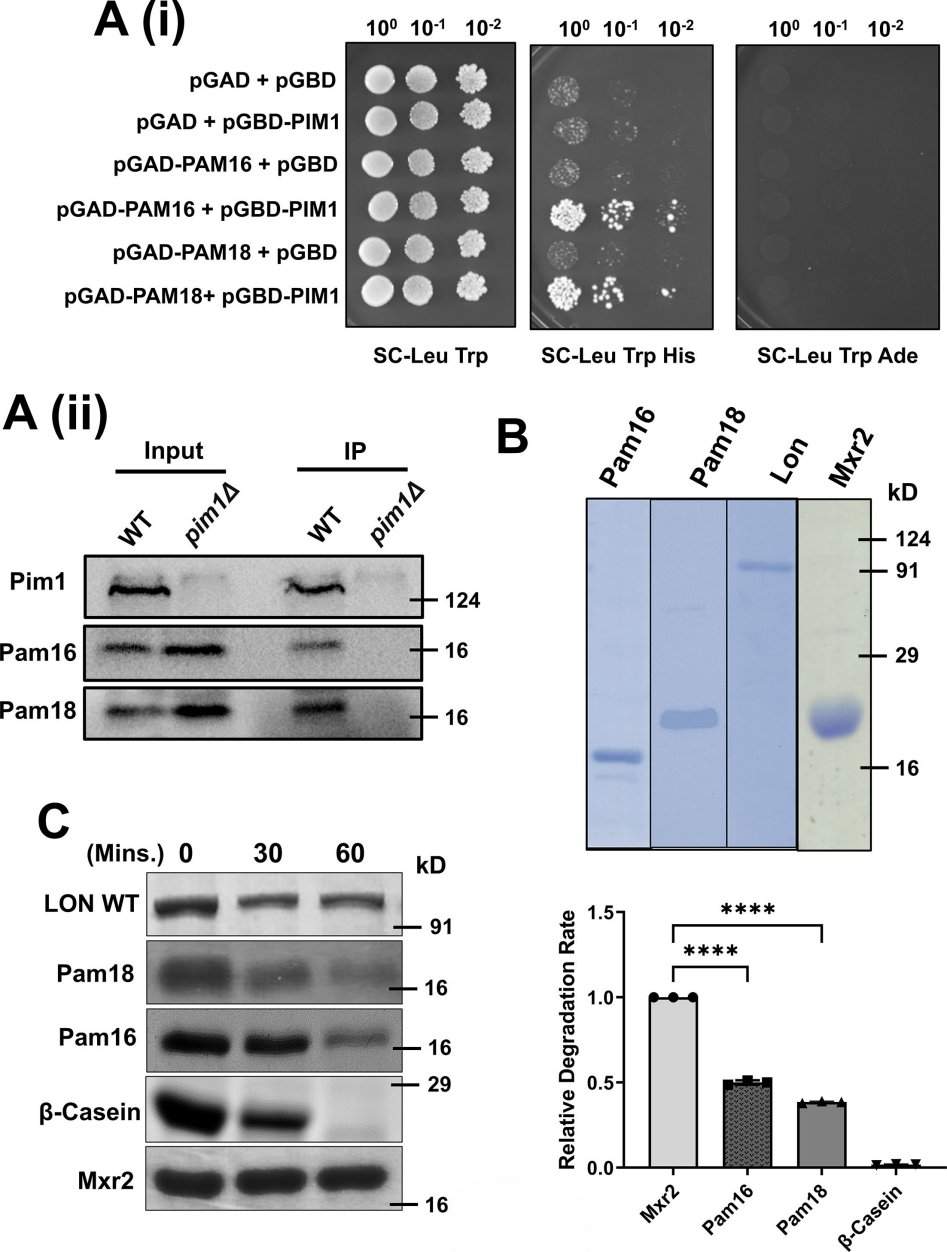
Lon protease degrades Pam16 and Pam18 *in vitro* **A.** Checking *in vivo* interaction between Pim1 and Pam16/Pam18. (**i**) Yeast two-hybrid assay. The PJ69-4A strain(, transformed with pGBD-C1-PIM1 and either pGAD-C1-PAM16 or pGAD-C1-PAM18 construct, was spotted along with control strains on agar plates of indicated minimal media. The growth profile was checked after incubation at 30°C for 2 days. (**ii**) Immunoprecipitation assay. Cell lysates from WT and *pim1Δ* cells were subjected to immunoprecipitation using Pim1 antibody. Input (10%) and elution fractions were then analyzed using SDS-PAGE followed by immunoblotting with Pim1, Pam16, and Pam18 antibodies. **B.** Purified recombinant proteins of yeast Pam16 (lane1), Pam18 (lane2), human Lon (lane3), and Mxr2 (lane4) were purified as described in materials and methods, resolved on SDS-PAGE and Coomassie stained. **C.** (Upper Panel) α-Casein (20 μg), Pam18, Pam16, and Mxr2 (5 μg) were incubated with human Lon WT (1 μg) in standard reaction conditions, as described in materials and methods. At indicated times, aliquots were removed, separated by SDS–PAGE and stained with Coomassie blue. α-Casein, known to undergo proteolysis by Lon protease, was used as a positive control, whereas Mxr2, which is not a substrate of Lon protease, was used as a negative control. (Lower Panel) Quantification of α-casein, Pam18, Pam16, and Mxr2 protein levels at 0 and 60 min. The amount of α-casein and Mxr2 was set to 1 (control). Amounts of full-length and leftover amount of protein after degradation were quantified by ImageJ and plotted as relative band intensity. The data shown are the mean ± SD of three biologically independent experiments. Statistical analysis was done using one-way ANOVA test (for C). ****P < 0.0001, ***P < 0.001, **P < 0.01, and *P < 0.05. ns, nonsignificant; Mxr2, methionine sulfoxide reductase 2.

If Pim1 is involved in the turnover of the components of the Pam complex, it may physically interact with these proteins. Therefore, we aimed to investigate whether Pim1 interacts with Pam16 and Pam18 using a yeast two-hybrid assay. In this assay, the PAM16 and PAM18 genes were cloned into the pGAD-C1 vector, while the PIM1 gene was cloned into the pGBD-C1 vector. The constructed plasmids were transformed, along with vector controls, into suitable yeast strains. The transformants were then grown on minimal media, serially diluted, and spotted on appropriate media plates (Figure 1A) before being incubated at 30°C for two days. The results of the yeast two-hybrid assay showed that transformants containing Pim1 with either Pam16 or Pam18 could grow in minimal media lacking histidine but not in media lacking adenine. These results suggest that Pim1 interacts with Pam16 and Pam18. However, the interaction is weak (Figure 1A(i)). To further confirm the interaction between the Pam18/Pam16 complex and Pim1, an immunoprecipitation experiment was performed in WT and *pim1Δ* cells. Cell lysates from these strains were immunoprecipitated using the Pim1 antibody, and the eluted fractions were analyzed by western blotting with Pam16, Pam18, and Pim1 antibodies. The immunoprecipitation assay results exhibited the presence of both Pam16 and Pam18 in the eluted fraction of only WT cells but not in *pim1Δ* cells, which confirms specific *in vivo i*nteraction between Pim1 and Pam16/Pam18 (Figure 1A(ii)).

Pim1 protease in *Saccharomyces cerevisiae* is known to undergo self-degradation and lose activity *in vitro* [59]. However, the human homolog of Pim1, Lon protease 1, has been successfully purified in its active form and used for several *in vitro* experiments [60]. Hence, we have used human Lon for *in vitro* degradation assays, considering that it has been tested and proved to be a substitute for Pim1 *in vivo* [61].

Lon protease, Pam18, Pam16, and Mxr2 (methionine sulfoxide reductase 2) recombinant proteins were expressed and purified for *in vitro* degradation assays. After confirming that purified Pam18, Pam16, and Mxr2 proteins were stable upon incubation at 30°C and 37°C, we mixed them with a substoichiometric concentration of Lon protease. We incubated them at 30°C for 30 and 60 min, separated them on SDS-PAGE, and Coomassie stained. The amount of Pam16 and Pam18 proteins in the reaction decreased significantly with time, and we observed ~2.5-fold degradation after 60 min of incubation (Figure 1B and C). Mxr2, a mitochondrial matrix protein used as a negative control, undergoes no degradation. α-Casein, an intrinsically unfolded protein known to be proteolytically degraded by Lon, is used as a positive control in this assay. These *in vitro* results suggest that Pam18 or Pam16 are probable substrates of Lon protease (Figure 1B & C).

### Lon-type protease Pim1 regulates Pam18 or Pam16 levels *in vivo*

**Figure 2:**
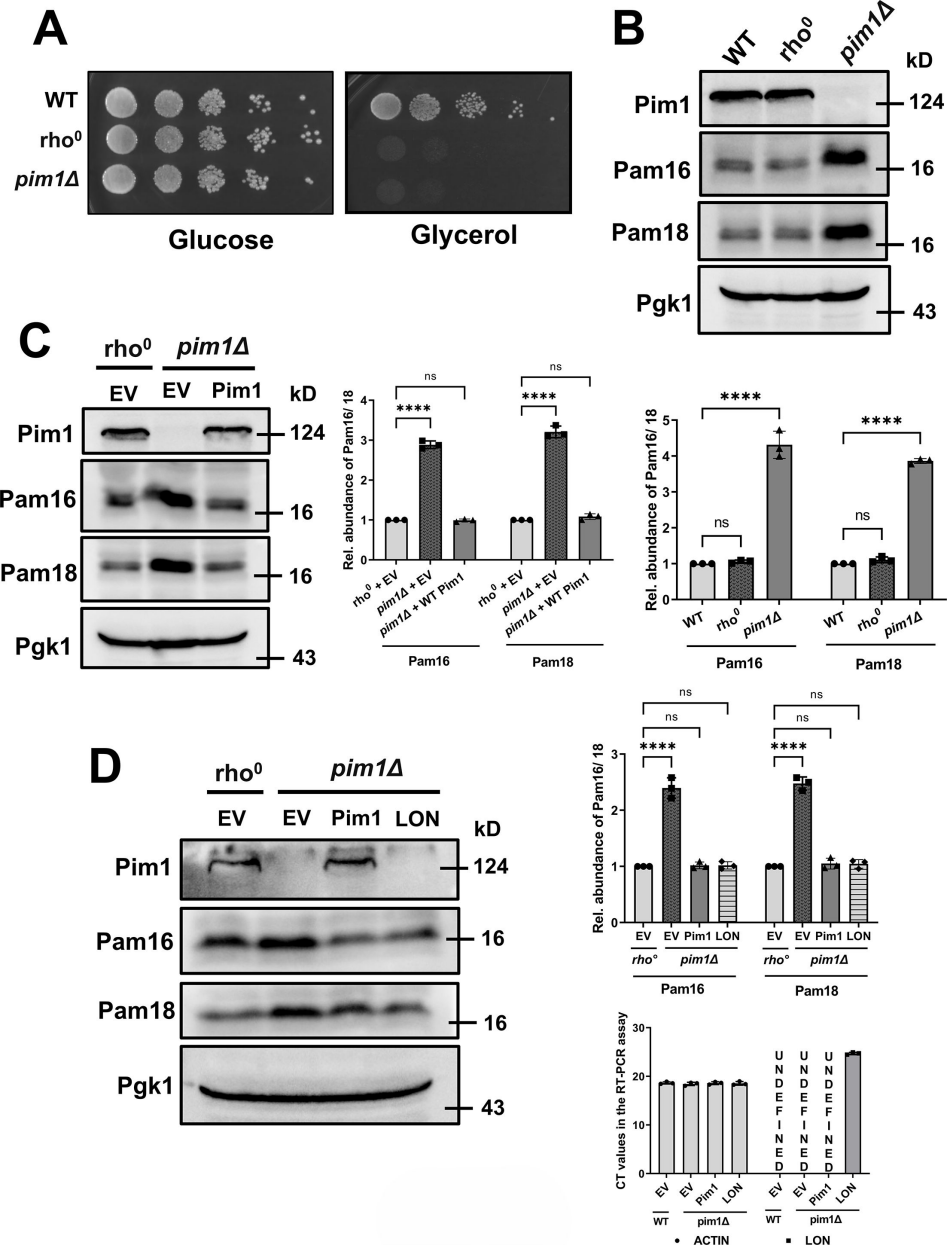
Accumulation of PAM complex proteins *in vivo* in *pim1Δ* **A.** Generation of *rho^0^* cells. WT cells were grown in YPD agar plates containing 25 µg/ml EtBr for two generations. After growth, the cells were tested for mitochondrial DNA depletion along with WT (positive control) and *pim1∆* (negative control) cells by growing them in YPD and YPG plates. After the spotting assay the plates were incubated at 30° for 2 days and growth profile was checked. **B.** Whole-cell extracts (from 1.0 OD cells) from WT, *rho^0^,* and *pim1Δ* were analyzed by SDS-PAGE and immunoblotted against indicated specific antisera. Pgk1, a cytosolic protein, serves as a loading control. Quantification of the protein abundance is represented in the lower panel. **C.** Whole-cell extracts (from 1.0 OD cells) from *rho^0^*, *pim1Δ,* and *pim1Δ*/Pim1 were analyzed by SDS-PAGE and immunoblotted against indicated specific antisera. Pgk1, a cytosolic protein, serves as a loading control. Quantification of the protein abundance is represented in the right panel. **D.** Whole-cell extracts (from 1.0 OD cells) from *rho^0^*, *pim1Δ*, *pim1Δ*/Pim1, and *pim1Δ*/Lon were analyzed by SDS-PAGE and immunoblotted against indicated specific antisera. Quantification of the protein abundance is represented in the upper right panel, while the Lon transcript level in the strains is represented in the lower right panel. The data shown are the mean ± SD of three biologically independent experiments. Statistical analysis was done using one-way ANOVA test (for B, **C, and D**). ****P < 0.0001, ***P < 0.001, **P < 0.01, and *P < 0.05. ns, nonsignificant. EV, empty vector control.

Next, to examine the regulation of Pam18 and Pam16 *in vivo*, we wanted to check the abundance of PAM complex proteins in WT and *pim1∆* cells. As *pim1∆* cells lack mitochondrial DNA, we want to check the abundance of PAM complex proteins in *rho^0^* cells. The WT cells were treated with ethidium bromide to generate *rho* ^*0*^ cells. WT *and pim1∆* cells were spotted on the Yeast Peptone Dextrose (YPD) or Yeast Peptone Glycerol (YPG) plate to confirm mitochondrial DNA depletion. The growth profile on these plates showed that, though the WT strain could grow on YPD and YPG plates, *rho^0^ and pim1∆* strains could not grow on the YPG plate, confirming mitochondrial DNA depletion in these cells (Figure 2A).

We wanted to check the steady-state levels of PAM complex proteins in *pim1∆* cells. WT, *rho^0^,* and *pim1∆* strains were grown in minimal media until the mid-log phase. Cell lysates from these strains were analyzed via immunoblotting analysis as described in the methods. The immunoblotting result showed that the Pam16 and Pam18 protein levels were markedly higher in the *pim1∆* strain than in WT and *rho^0^* strains. On the other hand, the abundance of these two proteins is comparable in both WT and *rho^0^* cells (Figure 2B). These results suggest that the increased abundance of Pam18 and Pam16 in *the pim1∆* strain is not due to mitochondrial DNA depletion but might be due to the absence of Pim1 protease.

To further confirm the involvement of Pim1 in the regulation of PAM complex proteins, *pim1∆* strains having either an empty vector or expressing Pim1 and *rho^0^* strain with an empty vector were subjected to immunoblot analysis as described in the methods. Western blot data shows that the enhanced levels of Pam16 and Pam18 in the *pim1∆* strain decreased upon expressing WT Pim1 in the strain (Figure 2C). Previous studies [62] demonstrated that the human Lon protease, a functional homolog of yeast Pim1, efficiently degrades the Pim1 substrate Isu1. To assess whether Lon similarly targets Pam16 and Pam18, we expressed Lon protease in the *pim1Δ* strain. Western blot analysis revealed that Lon expression markedly reduced the elevated levels of Pam16 and Pam18, comparable to WT Pim1 activity, indicating that Lon can substitute for Pim1 in regulating these substrates (Figure 2D). These results confirm the involvement of Pim1 in the regulation of Pam16 and Pam18 steady-state levels.

### Pim1 is specifically involved in the degradation of Pam18 or Pam16

**Figure 3:**
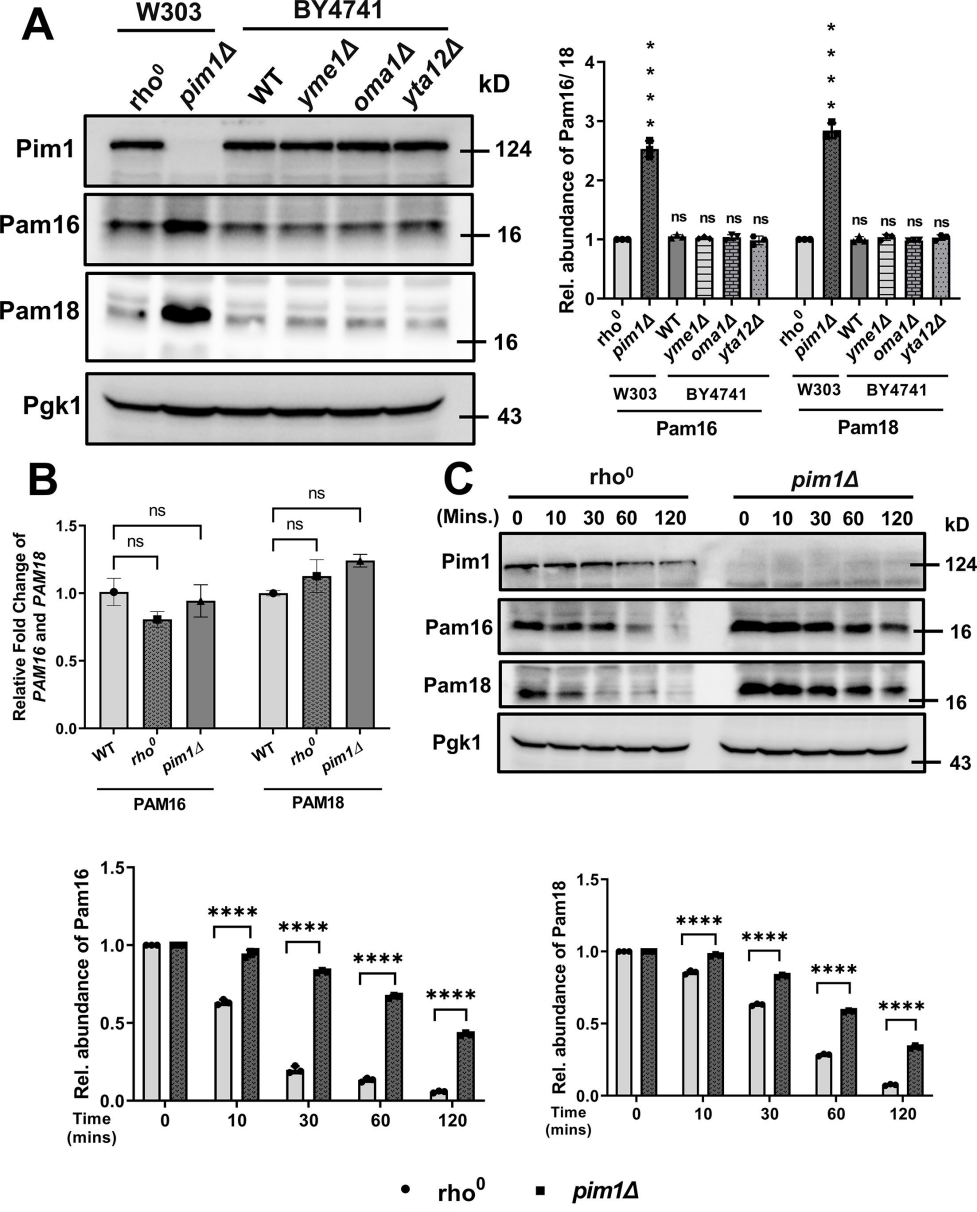
Pim1 specifically degrades PAM complex proteins *in vivo.* **A.** Whole-cell extracts (from 1.0 OD cells) from *rho^0^*, *pim1Δ* (W303 background) WT, *yme1Δ, oma1Δ,* and *yta12Δ* were analyzed by SDS-PAGE and immunoblotted against indicated specific antisera. Pgk1, a cytosolic protein, serves as a loading control. Quantification of the protein abundance is represented in the right panel. **B.** Whole RNA samples were isolated from WT, *rho^0^,* and *pim1Δ* cells and used for qRT-PCR analysis to quantify the PAM16 and PAM18 expression. **C.** Cycloheximide chase assay between *rho^0^* and *pim1Δ* strain. After growing to mid-log phase, cells were treated with 50 µg/ml cycloheximide for the indicated time points and analyzed by SDS-PAGE and immunoblotted against the indicated specific antisera. Pgk1, a cytosolic protein, serves as a loading control. Quantification of the protein abundance is represented in the down panel (on left Pam16 and on right Pam18). The data shown are the mean ± SD of three biologically independent experiments. Statistical analysis was done using one-way ANOVA test (for A, **B, and C**). ****P < 0.0001, ***P < 0.001, **P < 0.01 and *P < 0.05. ns, nonsignificant; qRT-PCR, quantitative real-time PCR.

Other proteases of the mitochondrial inner membrane, known to have a wide range of substrate specificities, might also be necessary for Pam18 and Pam16 degradation. To check this, we measured Pam18 and Pam16 levels in *yta12Δ* and *oma1Δ* cells, which lack m-AAA and Oma1 proteolytic activity, respectively. As controls, we included a deletion of *yme1Δ*, which encodes the i-AAA protease whose active site faces the IMS, and a deletion of PIM1. Only the *pim1Δ* cells have elevated Pam18 and Pam16 levels compared with WT/*rho^0^* cells (Figure 3A), while other protease deletion mutants show comparable abundance of Pam18 and Pam16-like WT/*rho^0^* cells. These results suggest the specificity of Pim1 protease in Pam18 and Pam16 turnover *in vivo*.

To assess whether increased abundance of Pam16 and Pam18 in the *pim1∆* strain is due to up-regulated expression of Pam16 or Pam18 transcript levels or more stability of the Pam16 and Pam18 proteins. To test this hypothesis, whole RNA was isolated from WT, *rho^0^,* and *pim1∆* strains, followed by complementaary DNA (cDNA) preparation, as described in materials and methods. Quantitative real-time PCR (qRT-PCR) results show no significant difference in the expression. Furthermore, a cycloheximide chase assay was performed (Figure 3B) to check the stability of Pam16 and Pam18 in *rho^0^* and *pim1∆* strains. Cycloheximide results show that, in the *rho^0^* strain, the turnover of both Pam16 and Pam18 proteins is significantly faster than in the *pim1∆* strain (Figure 3C). These results suggest that the Pim1 protein is specifically involved in the turnover of the Pam16 and Pam18 proteins.

### The proteolytically active Pim1 is critical for the degradation of Pam18 and Pam16

**Figure 4:**
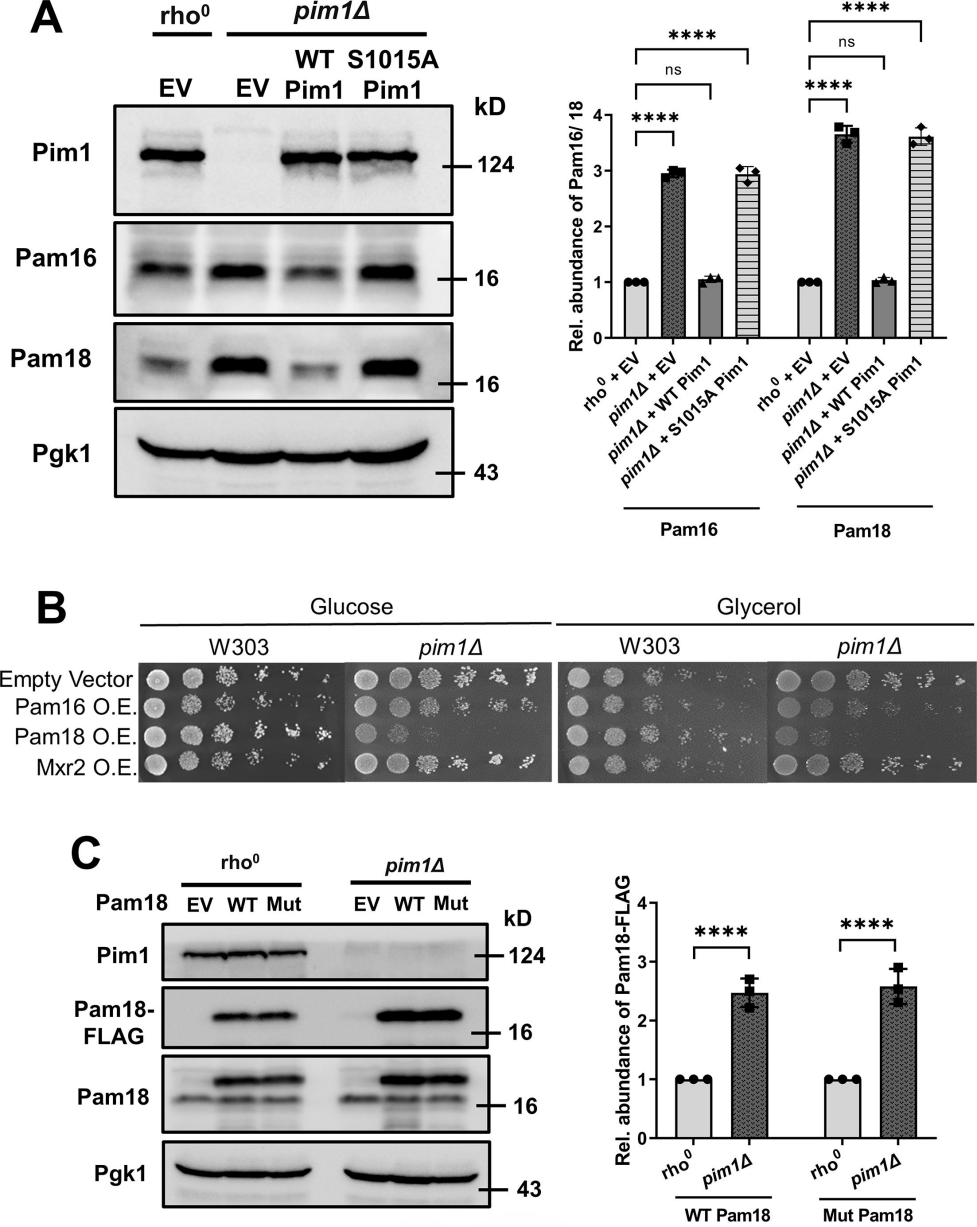
The proteolytic active Pim1 is critical for the degradation and steady-state level maintenance of Pam18 or Pam16. **A.** Whole-cell extracts (from 1.0 OD cells) from *rho^0^*, *pim1Δ*, *pim1Δ*/Pim1, and *pim1Δ*/Pim1S1015A were analyzed by SDS-PAGE and immunoblotted against indicated specific antisera. Pgk1, a cytosolic protein, serves as a loading control. Quantification of the protein abundance is represented in the right panel. **B.** WT and *pim1*Δ strains transformed with indicated plasmids were grown till log phase, equalized to OD 1 and plated on glucose and glycerol + 0.2% glucose containing medium as 1:10 serial dilution. Plates were incubated at 30°C for 2 days. **C.** Whole-cell extracts (from 1.0 OD cells) from *rho^0^*, *pim1Δ* cells either having empty vector or overexpressing FLAG tagged WT or mutant Pam18 (Δ102–104) cells were analyzed by SDS-PAGE and immunoblotted against indicated specific antisera. Pgk1, a cytosolic protein, serves as a loading control. Quantification of the protein abundance is represented in the right panel. EV, empty vector control.

Our *in vitro* and *in vivo* results suggest that Pam18 and Pam16 undergo degradation by Pim1. Pim1 has different domains for different functions [63]. Therefore, we asked whether its proteolytic activity is critical for regulating Pam18 and Pam16 levels *in vivo*. We took advantage of the Pim1 variant, which is proteolytically inactive due to substituting the catalytic serine Ser-1015 by alanine (Pim1 S_A) but maintains its other functions. We compared Pam16 and Pam18 levels in *pim1Δ* cells harboring an empty vector or vectors containing PIM1 WT or pim1 S_A to that in WT cells (Figure 4A). As expected, Pim1 WT expression resulted in reduced levels of Pam18 and Pam16, whereas expression of Pim1 S_A did not.

To test whether Pim1 is required to maintain steady-state levels of Pam16 and Pam18, we overexpressed Pam16, Pam18, and Mxr2 in WT and *pim1∆* strains, and a growth assay was performed on YPD and YPG agar plates. Since *pim1∆* is a rho- and displays minimal growth on non-fermentable carbon sources, we supplemented 0.2% glucose to the YPG plates. Overexpressing Pam16 and Pam18 did not affect WT in either glucose or glycerol media, but there was a decreased growth in *pim1∆*, which was more prominent in Pam18 overexpression (Figure 4B). Our results suggest that Pim1 is required to maintain a stoichiometric ratio of Pam16 and Pam18.

To assess whether Pim1-mediated degradation of Pam16 and Pam18 depends on the stability of their heterodimeric interaction via the J- and J-like domains, we overexpressed WT Pam18 and a mutant variant (Δ102–104) known to disrupt heterodimer formation in *rho⁰* and pim1Δ strains [29,30]. The steady-state levels of both WT and mutant Pam18 were examined by western blotting in *rho⁰* and *pim1Δ* cells. The results showed no significant difference in Pam18 levels between the WT and mutant forms within each strain. However, both variants accumulated to higher levels in *pim1Δ* cells compared with *rho⁰* cells. These findings indicate that Pim1-dependent degradation of Pam16 and Pam18 occurs independently of the stability of the Pam16–Pam18 heterodimer.

## Discussion

Mitochondrial proteases emerge as central regulators that regulate the quality and function of imported proteins. Improper mitochondrial proteostasis has been linked with severe human diseases, cancer, and neurodegenerative disorders [64–67]. ATP-dependent proteases are key regulators of mitochondrial quality control and its various functions. Pim1, a Lon-like serine protease in *S. cerevisiae*, was previously known to degrade ^3^H-labeled casein *in vitro* and be involved in the regulated turnover of specific mitochondrial proteins. It is involved in the ATP-dependent proteolytic breakdown of misfolded proteins and protein turnover in the mitochondrial matrix [52,55,68]. Most proteins are translocated to the mitochondrial matrix through the TIM23 complex. The PAM motor drives the import of pre-proteins through TIM23 translocase. To maintain a healthy mitochondrial proteome, the turnover of proteins constantly engaged in the import process must be adequately taken care of. How inner mitochondrial membrane AAA+ proteases regulate PAM complex protein turnover has yet to be studied. Since mitochondrial Hsp70 co-operates with Pim1 protease, we speculated that Pim1 might regulate the turnover of PAM complex proteins.

Aberrant protein accumulation in mitochondria is associated with human diseases, including Parkinson’s and Huntington’s [69]. Pim1 is an integral part of the cellular quality control process that promotes protein folding and prevents aggregation by binding and shielding hydrophobic peptide segments of client proteins. Pim1 decides the fate of the client protein by binding and holding the misfolded protein, which either undergoes proper folding with the help of other chaperones or gets degraded [37,38]. However, the role of Pim1 in regulating the physiological processes by degrading specific proteins needs to be explored [50,54]. Pam16 and Pam18 co-ordination is essential to fine-tune the import process in mitochondria and regulate the assembly of respiratory subunits [70,71]. The absence and accumulation of these proteins can be toxic to cells. Hence, the steady-state levels of these proteins are maintained constantly inside the mitochondria. In our study, we establish the specific role of Pim1 protease in regulating these PAM subunits.

We first demonstrated that the mitochondrial proteins Pam16 and Pam18 are substrates of the Pim1 protease, as both accumulate in its absence. Although we cannot fully exclude the possibility of partial unfolding during *in vitro* assays, our *in vivo* data strongly support that Pam16 and Pam18 are bona fide Pim1 substrates. Membrane-integrated proteases such as m-AAA and i-AAA are known to degrade mitochondrial membrane proteins [70]. However, deletion of these proteases did not significantly alter Pam16 or Pam18 levels. This suggests that their turnover is primarily regulated by Pim1, rather than by other inner membrane proteases. Furthermore, a Pam18 J-domain mutant (Δ102–104), which disrupts heterodimer stability with Pam16, did not show reduced protein levels in the presence of Pim1, indicating that degradation by Pim1 is independent of J-domain–mediated heterodimer formation (Figure 4C). Nonetheless, we cannot rule out the possibility of an indirect role for Pim1. It may influence the stability of Pam16 and Pam18 by regulating factors required for their proper assembly or maintenance. Thus, loss or inactivation of Pim1 could destabilize these proteins indirectly. Further studies are required to define the physiological conditions under which Pim1 mediates the degradation of Pam16 and Pam18.

Further, we showed that the proteolytic domain of the protease carries out the degradation process. Also, the accumulation of PAM subunits has a deleterious effect on cells without Pim1 protease (Figure 4). Hence, Pim1 is likely an intrinsic regulator for Pam18 and Pam16 subunits and extends the repertoire of substrates of PIM to regulate mitochondrial proteostasis.

## Materials and methods

### Yeast strains and plasmids

Yeast strains used in this study were derivatives of W303 or BY4741, as listed in Table 1. Standard yeast protocols were used for culturing yeast strains [73]. Yeast cells were grown in YPD (1% yeast extract, 2% peptone, and 2% dextrose) medium or synthetic minimal medium with dextrose minimal medium that contained 0.7% yeast nitrogen base with auxotrophic amino acids and 2% glucose. For the growth assay, 1 OD of cells was serially diluted on YPD or YPG (0.2% glucose) and incubated for 3 days at 30°C.

**Table 1:** Strains used in the study

Strain	Genotype	Reference
W303	MATa ;leu2- 3112 trp1-1 can1-100 ura3-1 ade2-1 his3-11	Euroscarf
BY4741	MATa ;his3Δ1 leu2Δ0 met15Δ0 ura3Δ0	Euroscarf
yNB111	W303 pim1∆::KANMX4	Euroscarf
yNB112	BY4741 pim1∆::KANMX4	Euroscarf
yNB185	BY4741 yme1∆::KANMX4	Euroscarf
yNB186	BY4741 yta12∆::KANMX4	Euroscarf
yNB187	BY4741 oma1∆::KANMX4	Euroscarf
yNB339	PJ469-A	(72)

The plasmids used in this study are listed in Table 2. For the construction of pET28a Pam16 and pET28a Pam18 plasmids, the coding sequence of Pam16 and Pam18 was PCR amplified from *S. cerevisiae* genomic DNA using primer pairs NB16/NB17 and NB14/NB15, respectively. The PCR product of Pam16 was digested with EcoRI/XhoI and Pam18 with BamHI/XhoI. These digested inserts were ligated into EcoRI/XhoI (for Pam16) and BamHI/XhoI (for Pam18) digested pET28a vector. The coding sequence of Pam16 and Pam18 was PCR amplified using primer pairs NB862/NB863 and NB864/NB865 to construct p425TEF Pam16 and p425TEF Pam18 plasmids. The PCR products were digested with BamHI/XhoI and were ligated into the similarly digested p425TEF vector. For construction of the pRS425 Pam18 WT 3X FLAG plasmid construct, the coding sequence of Pam18 was PCR amplified from *S. cerevisiae* genomic DNA using primer pairs NB16/NB17 and NB2181/NB2162 followed by digestion with SpeI/PstI and ligation within the digested pRS425 vector. On the other hand, for pRS425 Pam18 (Δ102-104) 3X FLAG construct, mutant Pam18 coding sequence was amplified from pNB1070 using same set of primers and then digested and ligated similarly within the digested pRS425 vectorThe primer sequence used in constructing these vectors is listed in Table 3. Yeast transformations were done using the standard lithium acetate method [72].

**Table 2:** Plasmids Used in the study

	Plasmid	Source/Reference
pNB460	pROEX LON WT	Carolyn Suzuki, Rutgers-New Jersey Medical School
pNB207	pET28 PAM16	This study
pNB144	pET28 PAM18	This study
pNB816	pET28 MXR2	(74)
pNB415	pGBD-C1	(73)
pNB422	pGAD-C1	(73)
pNB983	pGAD-PAM16	This study
pNB984	pGAD-PAM18	This study
pNB985	pGAD-PIM1	This study
pNB819	p414-ADH	(75)
pNB817	p414-ADH PIM1	(61)
pNB818	p414-ADH PIM1 mutant	(61)
pNB557	p414-ADH LON	(75)
pNB814	p425 TEF PAM16	This study
pNB815	p425 TEF PAM18	This study
pNB1068	pTEF leu Pam18 WT 3X Flag	This study
pNB1069	pTEF leu Pam18(Δ102–104) 3X Flag	This study
pNB1070	pRS315 Pam18(Δ102–104)	Patrick D’Silva, Department of Biochemistry, IISc, India.

**Table 3:** Primers

Primer name	Sequence (5′–3′)	Restriction enzyme
NB14 PAM18 F	CCCAGGATCCACCATGAGTTCTCAAAGTAATACTGGT	BamHI
NB15 PAM18 R	ATTCTCGAGTTATTTGCTAATACCCCTTTTTTCCAA	XhoI
NB16 PAM16 F	CCCAGAATTCACCATGGGAAAAGGCGAAGAATATGGTGGT	EcoRI
NB17 PAM16 R	ATTCTCGAGCTACTGATTGCTGCTTGCACTATT	XhoI
NB862 PAM16 F	AAA GGATCC ATGGCTCACAGGGCTTTCATAC	BamHI
NB863 PAM16 F	AAA GGATCC ATGGCTCACAGGGCTTTCATAC	BamHI
NB864 PAM16 F	AAA CTCGAG CTACTGATTGCTGCTTGCACTA	XhoI
NB865 PAM18 F	AAA GGATCC ATGAGTTCTCAAAGTAATACTG	BamHI
NB866 PAM18 R	AAA CTCGAG TTATTTGCTAATACCCCTTTTT	XhoI
NB1022 PAM16 R	GCC GTCGACCTACTGATTGCTGCTT	SalI
NB1018 PAM18 R	GCC GTCGACTTTGCTAATACCCCTTTTT	SalI
NB1102 PIM1 F	CTACCCGGGATGCTAAGAACAAGAACC	XmaI
NB1103 PIM1 R	ATGGTCGACTTAGTCCTTTTCCTTTTTAGC	SalI
NB2181 PAM18 F	AAAACTAGTATGAGTTCTCAAAGTAATACTG	Spe1
NB2162 PAM18 3XFLAG R	ATGCTGCAGTTACTTGTCATCGTCATCCTTGTAATCGATGTCATGATCTTTATAATCACCGTCATG GTCTTTGTAGTCGGATCCTTTGCTAATACCCCTTTTTTC	Pst1

### Expression and purification of recombinant proteins from *Escherichia coli* cells

pProEX Lon, pET28a Pam18, pET28a Pam16, and pET28a Mxr2 plasmids each were transformed to Rosetta (DE3) competent cells (Novagen). A single colony was inoculated into LB medium (1% bacto-tryptone, 0.5% yeast extract, 1% NaCl) containing ampicillin (100 µg/ml) or kanamycin (25 µg/ml) antibiotic and induced with 1 mM isopropyl thiogalactoside at 30°C for 2 hr by shaking at 220 rpm speed. Cultures were centrifuged at 8,000 rpm for 10 min and resuspended in buffer (25 mM Tris-HCl pH 8.0, 100 mM NaCl, and 2 mM DTT) and stored at −80°C. Cell suspensions were thawed to 4°C, and the suspension was lysed by extensive sonication for 20 cycles (15 sec ON and 45 sec OFF) at 30% amplitude. The bacterial lysates were cleared at high speed for 30 min, and the supernatant was loaded into Ni-NTA beads (Clontech, USA). Beads were washed with 3–5X volume of buffer containing 20 mM imidazole. Finally, proteins were eluted with a buffer containing 400 mM imidazole and dialyzed against a buffer containing 10% glycerol without imidazole. Protein aliquots were subjected to snap freeze using liquid nitrogen and stored at −80°C.

### *In vitro* Lon degradation assays

To monitor the ATP-dependent proteolytic activity of Lon, we monitored the degradation of α-casein (20 μg; Sigma Aldrich) as a control. Aliquots of purified proteins (5 µg) were incubated with 1 μg Lon in 50 mM Tris HCl, 100 mm KCl, 40 mm MgCl2, 1 mM DTT, and 5 mM ATP in a 50-μl reaction mixture. The degradation assays were carried out at 37°C at different time points (0, 30, and 60 min). After adding the enzyme to the protein, 2X loading dye was immediately added, kept at 4°C, and taken as 100% or zero time point. The samples were separated on SDS-PAGE and stained with Coomassie Brilliant Blue.

### Preparation of yeast cell lysates

Cells were grown overnight in a 10-ml appropriate selective medium to prepare crude cell lysates. Cultures were centrifuged at 5,000 rpm for 5 min (Eppendorf, Germany) at room temperature, and cells were washed with sterilized water and resuspended in 50 µl buffer (50 mM Tris-HCl pH 7.5, 100 mM NaCl, 1 mM PMSF (phenylmethylsulfonyl fluoride)) along with 0.2 g acid-washed glass beads. This mixture was vortexed vigorously and centrifuged at 13,000 rpm for 10 min at 4°C. The protein concentration in the supernatant was estimated using a Bradford protein assay kit (AMRESCO). In total, 50–100 µg of protein samples were separated on SDS-PAGE, transferred to a nitrocellulose membrane, probed with antibodies, imaged by Bio-Rad VersaDoc, and analyzed by Image J.

### Cycloheximide chase

Appropriate strains were grown to the mid-log phase in a synthetic complete medium and then harvested to perform a cycloheximide chase assay. Harvested cells were then treated with cycloheximide [SigmaAldrich, C7698] (50 μg/ml) for different time durations (0, 10, 30, 60, and 120 min). Cells were harvested after each time point, and whole cell lysate was prepared to analyze the protein degradation profile of Pam16 and Pam18 protein by immunoblotting.

### RNA extraction and qRT-PCR

As described [74], the whole cell RNA sample was isolated from the harvested via the hot phenol method. Extracted RNA samples were subjected to DNase digestion, followed by cDNA synthesis as described earlier [75]. Relative RNA abundance was measured by qRT-PCR using the PowerUp SYBR Green PCR Master mix (Applied Biosystems, A25742) via a Quanstudio 3 Real-Time PCR System.

### Yeast two-hybrid assay

For yeast two-hybrid assay, the respective GAL4 activation domain (pGAD) and GAL4 DNA-binding domain (pGBD) clone constructs were transformed within the PJ469-A strain along with proper vector controls. Transformants were grown up to mid-log phase, serially diluted, and spotted in agar media plates, as described in [76]. After spotting, media plates were incubated at 30°C for 2 days. After incubation, the growth profile was checked to determine the interaction between the proteins of interest.

### Co-immunoprecipitation assay

Cell extracts for co-immunoprecipitation were prepared as described previously [75]. Following protein quantification, 1 mg of cell lysate was incubated with Protein A/G PLUS-Agarose beads (Santa Cruz Biotechnology, SC-2003) at 4°C for 2 hr for preclearing. The precleared lysate was then incubated overnight at 4°C with the Pim1 antibody. Subsequently, the lysate and antibody mixture was incubated with Protein A/G PLUS-Agarose beads for an additional 4 hr to facilitate antibody binding. After incubation, the beads were collected, washed three times, and subjected to western blot analysis using Pim1, Pam16, and Pam18 antibodies.

### Statistics

Statistical analysis was performed using one-way ANOVA test (mentioned in the figure legends). The significance of differences was indicated based on the *P* value of the respective statistical tests, which is as follows: ****P<0.0001, ***P<0.001, **P<0.01, and *P<0.05. ns, nonsignificant. The values represented in the data quantification are the mean of three independent experiments, and the error bars shown in the graph indicate the standard deviation among the experiments.

## Open Access

This article has been published open access under our Subscribe to Open programme, made possible through the support of our subscribing institutions, learn more here: https://portlandpress.com/pages/open_access_options_and_prices#conditional

## Abbreviations

AAA+: ATPases Associated with diverse cellular Activities
IMS: intermembrane space
Mxr2: methionine sulfoxide reductase 2
PAM: presequence-associated motor complex
Pim1: ATP-dependent Lon Protease
TIM: translocase of inner membrane
TOM: translocase of outer membrane
mtHSP70: mitochondrial Hsp70
